# A novel flow cytometric method for enhancing acute promyelocytic leukemia screening by multidimensional dot-plots

**DOI:** 10.1007/s00277-019-03642-w

**Published:** 2019-03-04

**Authors:** Bettina Kárai, Mira Habók, Gyula Reményi, László Rejtő, Anikó Ujfalusi, János Kappelmayer, Zsuzsanna Hevessy

**Affiliations:** 1 Department of Laboratory Medicine, University of Debrecen, Nagyerdei krt. 98, Debrecen, H-4032 Hungary; 2 Department of Internal Medicine, Faculty of Medicine, University of Debrecen, Debrecen, Hungary; 3 Department of Hematology, Jósa András County Hospital, Nyíregyháza, Hungary

**Keywords:** Acute promyelocytic leukemia, Flow cytometry, Multidimensional dot-plot, Cryptic translocation

## Abstract

Acute promyelocytic leukemia (APL) is generally characterized by t(15;17)(q24;q21). In some cases, the classic translocation cannot be identified by conventional methods, since the *PML-RARA* fusion protein results from complex, variant, or cryptic translocation. The diagnostic algorithm of APL starts with screening methods, such as flow cytometry (FC), followed by fluorescence in situ hybridization or polymerase chain reaction to confirm the diagnosis. Our aim was to develop a novel protocol for analyzing APL samples based on multidimensional dot-plots that can provide comprehensive information about several markers at the same time. The protocol included four optimized multidimensional dot-plots, which were tested by retrospective reanalysis of FC results in APL (*n* = 8) and non-APL (*n* = 12) acute myeloid leukemia (AML) cases. After predicting the potential position of hypergranular- and microgranular-type aberrant promyelocytes, the percentages of blast populations were examined within the gates in all AML cases. The percentage of blasts in each predefined gate was well above the cut-off value (95%) in APL cases in all tubes. In non-APL AML cases, the percentage of blasts in the same gates never reached the cut-off value in all investigated tubes, and even when it did in a single tube, the pattern was markedly different from that observed in APL cases. In conclusion, multidimensional dot-plots can be used for screening APL even in cryptic APL cases, although reproducibility across several laboratories would require standardization of antibodies and fluorochromes. This easy-to-use and quick method can support the diagnosis of APL and the prompt initiation of the appropriate treatment.

**Electronic supplementary material:**

The online version of this article (10.1007/s00277-019-03642-w) contains supplementary material, which is available to authorized users.

## Introduction

Genetic alterations determine the biological behavior of acute myeloid leukemia (AML); therefore, these are the most effective independent prognostic factors and serve as the basis of classifications and guidelines [1, 2]. The recommendation of the World Health Organization (WHO) classifies AML according to recurrent genetic abnormalities, which are associated with specific clinicopathological features. Acute promyelocytic leukemia (APL) is mostly caused by the *PML-RARA* fusion protein [3]. The promyelocytic leukemia (*PML*) gene is located on chromosome 15, while retinoic acid receptor alpha (*RARA*) on chromosome 17 [1]. The balanced reciprocal classic t(15;17)(q24;q21) translocation is detected in 90–92% of APL patients [4, 5]. In the remaining cases, complex translocations involving chromosomes 15 and 17 or a submicroscopic insertion result in the rise of *PML-RARA* transcript [1]. On the basis of the morphological appearances of aberrant promyelocytes, APL can be classified as hypergranular or microgranular [1]. The release of procoagulant mediators from the leukemic cells is likely the most important mechanism, which is responsible for APL-associated coagulopathy. Disseminated intravascular coagulopathy (DIC) and systemic fibrinolysis, which usually occurs at the time of diagnosis, are the major causes of early death [6–8]. The immediate administration of retinoid differentiating agents, such as all-trans retinoic acid (ATRA) and arsenic trioxide, reduce the hemorrhagic complications of APL; moreover, chemotherapy completed with ATRA improves overall survival [9–13]. Therefore, rapid diagnosis and prompt treatment are indispensable. The diagnostic algorithm of APL starts with morphology and immunophenotype examinations [2]. Classic APL is characterized by a distinct morphology; yet, the microgranular type can mimic acute monoblastic leukemia, where the clinical history also resembles APL regarding coagulopathy [1, 14]. The common immunophenotypic alterations in APL, such as CD117, CD64, cytoplasmic MPO, CD33 bright expression, and loss or only weak intensity of CD34 and HLA-DR expression, have been known for decades; however, these are not specific for APL [1]. The morphology and immunophenotype examination serve as screening, and the detection of t(15;17) confirms the diagnosis. Fluorescence in situ hybridization (FISH) is the most commonly used method for the identification of t(15;17). In rare cases of APL that do not harbor the classic cytogenetically visible translocation but still possess the *PML-RARA* rearrangement, the polymerase chain reaction (PCR) is crucial for detecting the fusion gene [4, 5, 15, 16].

Due to the use of multiple lasers and an increasing number of fluorochromes, more information can be obtained from cells by flow cytometric examinations, which lead to the increasing significance of this method. Our aim was to exploit the opportunities afforded by recent improvements in analysis software, which can handle such large amounts of data. We wanted to assess a new analysis protocol based on multidimensional radar dot-plot that was designed to expand the effectiveness of flow cytometric examination in the screening of APL. To test this protocol, we compared the results of an APL AML group to those of a non-APL AML group with the help of predefined gates around the blasts characterized by the most common immunophenotype in APL.

## Material and methods

### Study design

We examined retrospectively the data of patients referred to the Department of Laboratory Medicine (University of Debrecen, Hungary) between May 2014 and December 2017 for detailed examination. On the basis of clinical history, morphological, flow cytometric, cytogenetic, and molecular examinations, two groups were formed: an APL group with eight patients and a non-APL group with 12 patients. Six patients with APL had classic t(15;17) translocation. One patient had complex karyotype affecting one additional chromosome beside chromosomes 15 and 17. Furthermore, one patient had cryptic APL, where the fusion gene could be detected only by PCR. The non-APL group was designed to include only those AML cases, which were characterized by myeloblasts that mimic the immunophenotype of APL. Their myeloblasts were CD117 positive, CD33 bright, and CD34 negative—this is the immunophenotype pattern most characteristic of APL. All patients in the non-APL group had normal karyotype and mutated *NPM1* because this genetic feature is associated with CD34 negative myeloblasts [17–20]. The clinical and laboratory parameters of patients are summarized in Table 1. Bone marrow aspiration samples were examined by May-Grünwald-Giemsa staining and ×1000 magnification.

**Table 1:** Clinical and laboratory parameters of patients

	APL (*n* = 8)	Non-APL (*n* = 12)
Age (year)	53 (32–74)	64 (50–80)
Gender (female/male)	4/4	9/3
WBC (×10^9^ L)	17.01 (0.46–56.1)	88.12 (8.3–303)
Hypergranular type	9.56 (0.46–22.45)	
Microgranular type	35.64 (15.2–56.1)	
HB (g/L)	96 (77–115)	81 (49–138)
PLT (×10^9^ L)	38 (8–85)	118 (28–274)
LDH (U/L)	806 (173–2266)	1345 (214–8194)
Blast% in bone marrow	63.5 (26.6–87.5)	54.8 (20.7–82.9)
DIC	8/8	0/12
*FLT3 ITD* (+/-/*n*)	3/3/2	4/8/0
*FLT3 TKD* (+/-/*n*)	0/6/2	0/12/0
*NPM1* (+/-/*n*)	0/6/2	12/0/0

Abbreviations: *WBC*: white blood cell count, *HB*: hemoglobin, *PLT*: platelet count, *FLT3 ITD*: *FLT3* internal tandem duplication, *FLT3 TKD*: *FLT3* tyrosine kinase domain, *NMP1*: nucleophosmin, *n*: not done

### Flow cytometry

The bone marrow samples were examined routinely by eight color-labeling procedure with a four-tube AML panel for diagnostic purposes. The antibodies we examined are shown in Table 2.

**Table 2:** Antibody combinations used in flow cytometric examination for the diagnosis of AML

	FITC	PE	PerCP-Cy5.5/PC5.5	PC7	APC	APC-AF750	PB	PO
1.	CD14	CD11b	HLA-DR	CD13	CD300e	CD64	CD4	CD45
2.	CD15	CD123	CD34	CD13	CD10	CD16	HLA-DR	CD45
3.	CD71	CD117	CD33	CD56	CD34	CD38	CD7	CD45
4.	cyFXIII-A	cyMPO	CD33	CD2	CD34	CD117	HLA-DR	CD45

Abbreviations: *cyFXIII-A*: cytoplasmic A subunit of blood coagulation factor XIII; *cyMPO*: cytoplasmic myeloperoxidase, *FITC*: fluorescein isothiocyanate, *PE*: phycoerythrin, *PerCP-Cy5.5*: peridinin chlorophyll protein 5.5, *PC5.5*: phycoerythrin cyanin 5.5, *PC7*: phycoerythrin cyanin 7, *APC*: allophycocyanin, *APC-AF750*: conjugation allophycocyanin-alexa fluor 750, *PB*: pacific blue, *PO*: pacific orange

CD14, CD11b, HLA-DR, CD45, CD64, CD13, CD15, CD34, CD71, CD117, CD300e, CD4, and CD10 markers were purchased from the Becton Dickinson Biosciences (San Jose, CA, USA); CD33, CD16,CD2, CD117, and CD13 markers were purchased from the Beckman Coulter (Brea, CA, USA); CD45 marker was purchased from Invitrogen (Thermo Scientific Inc., Waltham, MA, USA); HLA-DR marker was purchased from Biolegend (San Diego, CA, USA), and cytoplasmic MPO (cyMPO) was purchased from Dako (Santa Clara, CA, USA). Generation and labeling of mouse monoclonal antibodies against FXIII-A subunit was carried out utilizing a FITC labeling kit (Sigma, St. Louis, MO) [21]. The labeling procedure was performed as previously described [22]. One hundred thousand events were acquired with the help of FACS Canto II flow cytometer (Becton Dickinson Biosciences, San Jose, CA, USA). To make the results comparable, the flow cytometer was calibrated daily, using cytometer setup and tracking fluorescent microbeads (Cat No. 641319, Becton Dickinson Biosciences, San Jose, CA, USA) and Autocomp software as recommended by the manufacturer. Data was analyzed by Kaluza Software version 1.2 (Beckman Coulter, Brea, CA, USA).

Bivariate dot-plots were used to analyze the detailed immunophenotype of leukemic cells. The threshold of positivity was set to > 10% positive leukemic cells for MPO and to > 20% for all other antigens, in accordance with the threshold conventions apparent in the literature [1, 23].

To create an analysis protocol for APL, one multidimensional radar dot-plot was optimized for each of the four tubes. The software allows selecting the number and the position of parameters in the radar dot-plot, which influence the appearance of blast population in the dot-plot. The optimization procedure was the following: First, the files of three AML patients characterized by different morphology (M2, M4, APL) were merged, and then the three blast populations were gated by CD45/SSC bivariate dot-plot. Subsequently the three different blast populations were presented in one radar dot-plot, and those parameters and locations were selected whereby the three populations differed from each other the most (for tube 1, these were SSC, CD4, CD64, CD11b, CD13, HLA-DR, CD14, CD300e, CD45; for tube 2, CD15, CD123, SSC, CD34, CD13, HLA-DR, CD45, for tube 3, CD34, CD117, CD56, CD45, CD33, SSC, and for tube 4, these were cyFXIII-A, cyMPO, HLA-DR, SSC, CD117, CD45). Finally, we merged all hypergranular APL cases to designate gates for the expected positions of 95% (cut-off value) of hypergranular APL blast populations. Because the location of microgranular-type APL differed from hypergranular cases, a microgranular gate could be defined on the basis of the two microgranular cases.

#### Chromosome analysis, FISH, and molecular analysis

G-banding was performed according to standard procedures on all samples of APL and non-APL patients. Karyotypes were described according to the International System of Human Cytogenetic Nomenclature. Fluorescence in situ hybridization was carried out on cell suspension samples used for chromosome analysis according to the manufacturer’s instructions, using *PML*/*RARA* DC, DF translocation probes (Metasystems, Altlussheim, Germany).

#### Statistical analysis

Considering the low number of samples median, 25th and 75th percentile values were used. Statistical analysis and the creation of figures were carried out using GraphPad Prism 6.0 (GraphPad Software, San Diego, CA, USA) statistical program.

## Results

### Morphological characterization of patients with APL and non-APL AML

In the APL group, two patients displayed microgranular- and six patients with hypergranular-type APL. The myeloblasts of three patients in the non-APL group were characterized by agranular cytoplasm, showing distinct blebs, or pseudopod formation. Two patients had “cup-like” blasts. According to the French-American-British (FAB) classification, five of the remaining cases exhibited M4 morphology, one M0/M1, and one M2. The representative morphologic appearance of blasts in the APL is shown on Online Resource 1, respectively.

### Immunophenotypic characterization of patients with APL and non-APL AML

In accordance with the findings of the morphological examination, the aberrant promyelocytes had high side scatter (SSC) in six cases (hypergranular type) and medium intensity SSC in two cases (microgranular type). The immunophenotypes of promyelocytes are summarized in Table 3. Aberrant promyelocytes were characterized by a frequently detected pattern in APL: in hypergranular cases, promyelocytes expressed only CD117 of blast markers, while they expressed all myeloid markers (CD33, CD13, cyMPO) with high intensity. Blasts were CD15 negative in all cases. Regarding leukemia-associated immunophenotype (LAIP) and prognostic markers, CD56 and CD2 were positive in one case, while cytoplasmic FXIII-A was positive in all cases.

**Table 3:** Antigen expression by leukemic cells

	APL		Non-APL AML
	hypergranular type (*n* = 6)	microgranular type (*n* = 2)	*n* = 12
CD117	100%	100%	100%
CD34	0%	100%	0%
HLA-DR	0%	0%	58%
CD33	100%	100%	100%
CD13	100%	100%	100%
cyMPO	100%	100%	83%
CD14	0%	0%	42%
CD11b	0%	0%	42%
CD64	50%	50%	42%
CD4	0%	0%	42%
CD15	0%	0%	42%
CD123	0%	100%	75%
CD56	17%	0%	25%
CD38	100%	100%	100%
CD7	0%	50%	25%
CD2	17%	100%	0%
cyFXIII-A	100%	100%	75%

In the non-APL group, myeloblasts (MB) were characterized by monocytic immunophenotype in five cases, in accordance with the morphological findings. CD123 and cyFXIII-A were positive in 75% of the cases (Table 3).

### Multidimensional map of APL

**Fig. 1:**
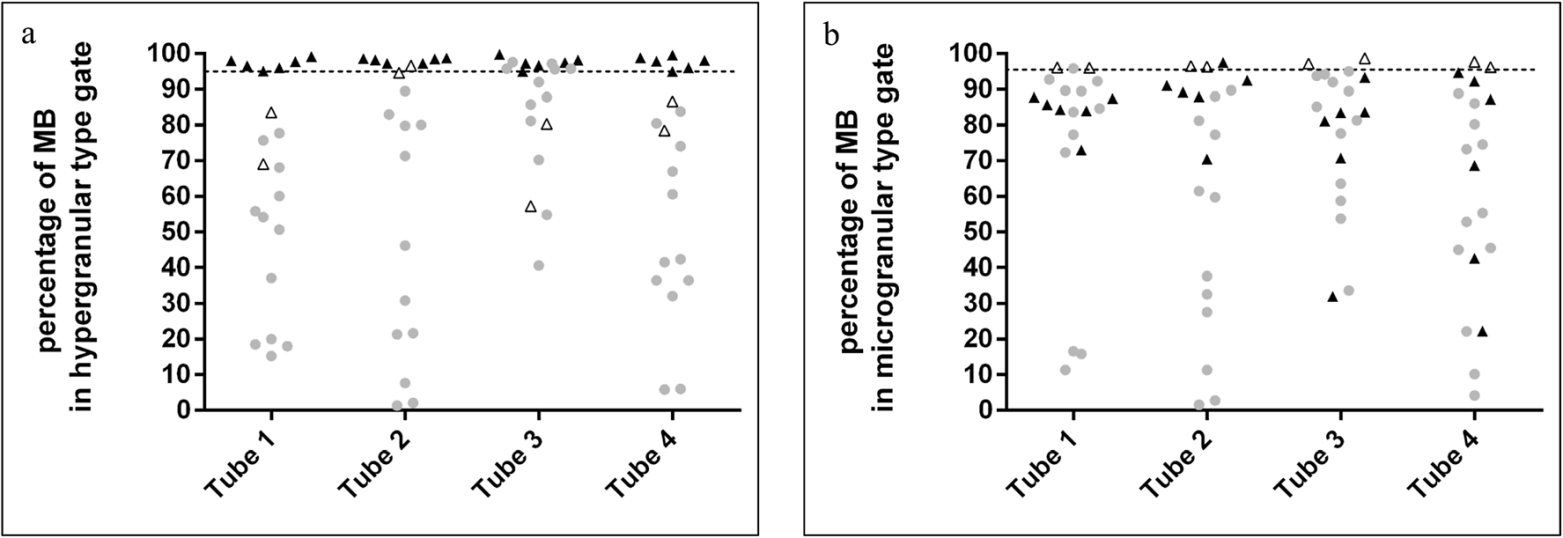
The percentage of blasts in hypergranular- (**a**) and microgranular-type gates (**b**). The black triangles indicate hypergranular APL cases, the white ones the microgranular-type APL cases, while the gray circles indicate non-APL AML cases. The horizontal line represents the cut-off value (95%)

**Fig. 2:**
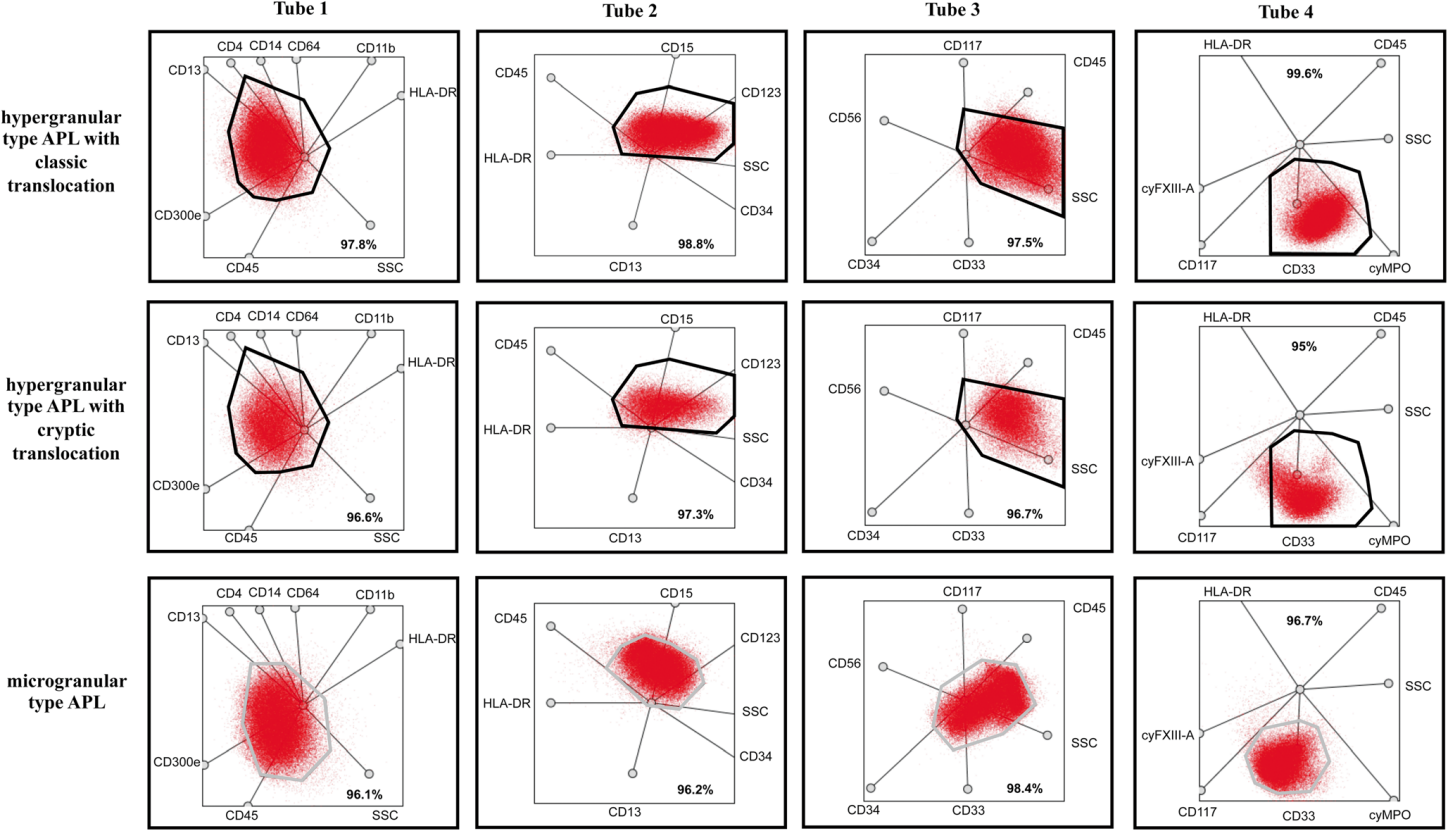
Representative dot-plots for various APL cases. Representative dot-plots of a hypergranular type APL with classic translocation are in the first line, dot-plots of hypergranular type APL with cryptic translocation are in the second, and microgranular type APL are in the third line. The bold frame indicates the hypergranular-gate, and gray one indicates the microgranular-gate. Red indicates blasts. The bold numbers indicate the percentage of blasts in gates predicted for one of the pre-defined type of APL-gate

Regardless of the mechanism that lead to the formation of the PML-RARA fusion protein, more than 95% of blasts detected in the hypergranular gate were observed in all hypergranular-type APL cases (Fig. 1a). As for the microgranular-type APL cases, the position of the blast population differed from what we found in the hypergranular-type APL cases; therefore, dedicated gates were set up to predict the position of microgranular blasts (Fig. 2). The microgranular blasts were located within their respective gate (the median percentages of blasts within the microgranular gate were 96.1% for tube 1, 96.5% for tube 2, 98% for tube 3, and 97% for tube 4). The percentage of blasts was well above not only the cut-off value (95%) in both hyper- and microgranular-type APL AML cases in all the four tubes (Fig. 1) but also the patterns of blasts within the gates corresponding to their types that were similar in all the cases.

**Fig. 3:**
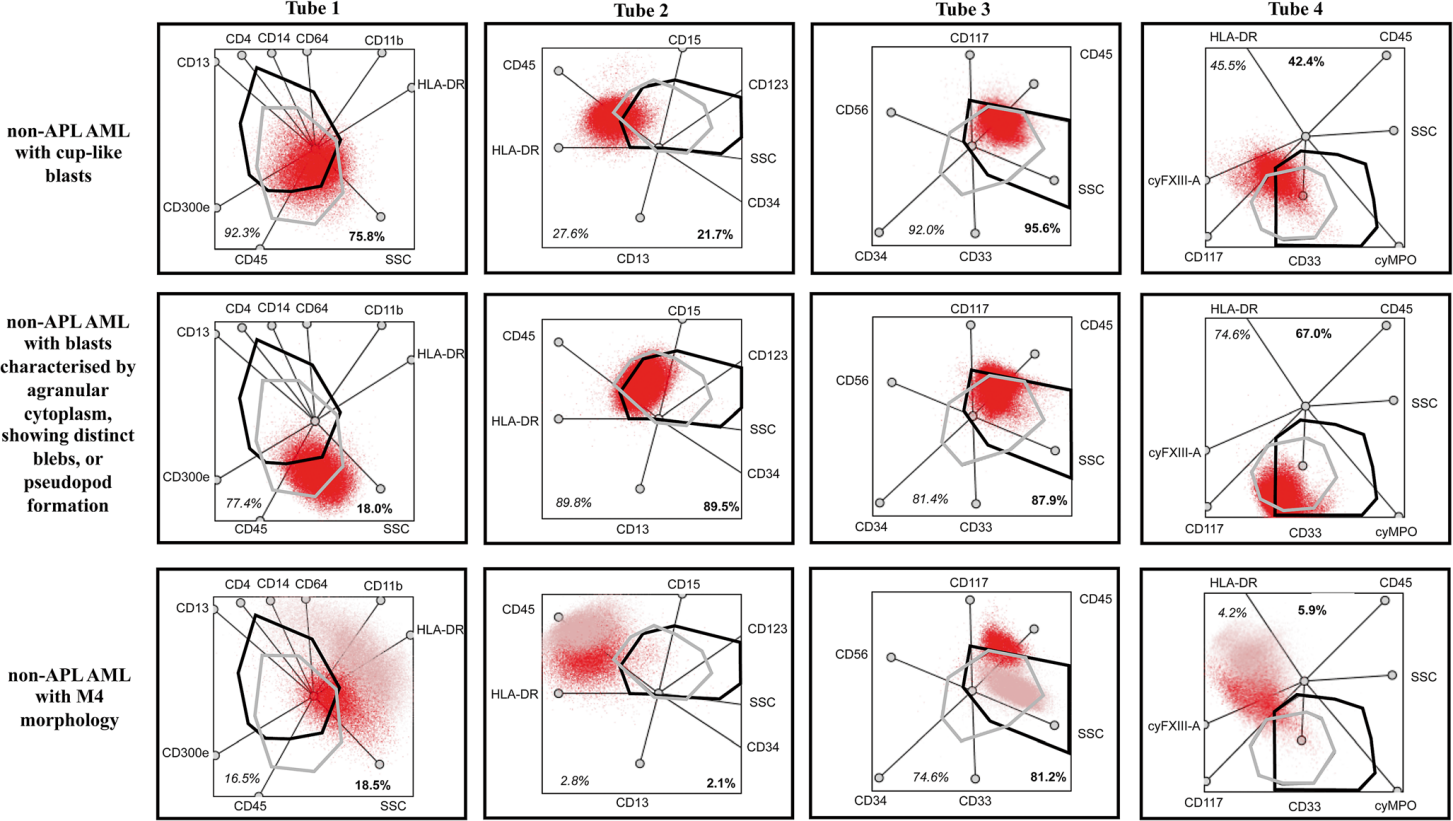
Representative dot-plots for various non-APL AML cases. Dot-plots of a non-APL AML with cup-like blasts are in first line, dot-plots of non-APL AML with blasts characterized by agranular cytoplasm, showing distinct blebs, or pseudopod formation are in the second and dot-plots of a non-APL with M4 morphology are in third line. The bold frame indicates the hypergranular-gate, and gray frame predicted microgranular-APL. Red indicates blasts; pink represents monocytes. The bold numbers indicate the percentage of blasts in gates predicted for hypergranular-type APL and represent the percentage of blasts in gates predicted for microgranular-type APL

In most cases of the non-APL AML patient group, blasts within the gate typically did not reach the cut-off value. It was only in tube 3—which contained the criteria markers (CD117, CD34) used for creating the non-APL AML group—that the percentage of blasts in the hypergranular gate surpassed the cut-off value (Fig. 1); but even here, the pattern of the blasts within the gate differed from what we detected in the APL AML group: the blasts were not dispersed evenly throughout the gate but concentrated in its upper segment (Fig. 2, tube 3). Similarly to the hypergranular APL gates, there were only one non-APL AML case which reached the cut-off value in the pre-defined microgranular gate in tube 1, and in some cases, the percentage of MB in the microgranular-type gate approached the cut-off value. The pattern of MB in the microgranular gate, however, differed from the position of microgranular APL cells. The representative multidimensional dot-plots in the APL and non-APL AML groups are shown in Figs. 2 and 3.

## Discussion

The major finding of our study was that with the help of predefined gates in a multidimensional radar dot-plot, an effective, reproducible, and quick protocol can be created for the screening of APL. Recently, routinely used flow cytometric analyzing software (e.g., Kaluza and Infinicyt) allows integrated data visualization, which improves the interpretation of a large amount of data generated by an increasing number of fluorochromes. The comprehensive dot-plots were used first in the identification of normal cell populations during maturation [24, 25]. When the position of normal cell types was mapped, on the one hand, the detection of alterations from the normal pattern permit the identification of characteristic dysplastic signs referred to as myeloproliferative disorders [24]. On the other hand, several studies have demonstrated that the appearance of aberrant cells on integrated dot-plots was associated with a distinct malignancy, which can be utilized in the detection of minimal residual disease [26–28].

To the best of our knowledge, we were the first to examine APL with multidimensional dot-plots based on a wide range of markers. We used not only the markers the expression of which was frequently altered in APL (CD34, CD117, HLA-DR, CD15, cyMPO, CD13, CD33, CD64) but also the prognostic markers, such as CD56, CD7, CD2, and cyFXIII-A [22, 29–32]. The expression of CD56 or CD7 marker is associated with poor prognosis [29], while the presence of cyFXIII-A or CD2 with good prognosis [22, 31].

We found that the aberrant promyelocytes can be characterized by unique patterns and positions on radar dot-plots regardless of the mechanism leading to the formation of PML-RARA fusion protein. Therefore, the results of these dot-plots can support the diagnosis of APL even in FISH-negative—cryptic—cases and indicate the performance of PCR examination as early as possible to confirm the diagnosis.

In addition, with the help of multidimensional dot-plots, more information can be obtained from blast cells with a single examination; therefore, APL cases can be differentiated from all types of AML, including CD34-negative cases. Earlier several studies confirmed the usefulness of bivariate dot-plots in the screening of APL cases within AML. These were based only on three or four markers, usually CD34, HLA-DR, and a marker indicative of maturation, such as CD15 or CD11b [30, 33, 34]. These bivariate dot-plots, however, were characterized by high specificity and sensitivity (> 90%) only with respect to all types of AML, most of which are CD34-positive cases. Our novel protocol based on multivariate dot-plots, in turn, brings the most value where the applicability of bivariate dot-plots is limited: differentiating between APL and AML with CD34- and/or HLA-DR-negative immunophenotype. More and more information has been accumulated over the past decade about this less common type of AML. It is characterized by normal karyotype, *NPM1* mutation, and morphologically by “cup-like” blasts and M2 or M4 morphology as defined by FAB [17–20, 35, 36]. Despite these common denominators, morphology suggests that this is not a homogeneous subtype of AML, and yet, another advantage of our protocol is that it can detect various patterns among CD34-negative non-APL cases. Thus radar dot-plots enable the more precise identification of similarly behaving AML cases.

It must be acknowledged that there are some limitations to this study. For three reasons, we were able to examine only a relatively small number of APL cases. First, APL is relatively uncommon in Hungary; secondly, radar dot-plots can be used to compare cases where the same antibodies and fluorochromes have been used; and thirdly, the eight-color staining method has been used in Hungary only since 2014. Therefore, a control study on a larger population would be necessary in order to validate our results. In addition, we examined only APL cases with *PML-RARA* fusion, but *RARA* may have other fusion partners, such as *ZBTB16*, *NUMA1*, *STAT5B*, or *NPM1*. The variant fusion partner is important because it can influence the prognosis through the response to ATRA. Sainty et al., who examined a large number of cases with APL lacking t(15;17), found that *ZBTB16-RARA* cases were associated with CD56 expression, and there were no other immunophenotypic differences from t(15;17) APL [37], which suggests that our multidimensional screening protocol could be used in those cases as well.

In conclusion, multidimensional (radar) dot-plots can be used for screening APL even in cryptic APL cases. Based on only four multidimensional dot-plots, our protocol examined 6–9 markers per tube at the same time, thus increasing the efficiency and effectiveness of the FC examination. This method is reproducible (within the same laboratory), easy-to-use, and quick regardless of the percentage of blasts size and can differentiate squarely between APL and non-APL AML cases even when the FC results based on bivariate dot-plots would be uncertain and suggest APL falsely. This differentiation is crucial, because APL requires the prompt administration of special treatment to achieve a favorable prognosis.

## Electronic supplementary material

Online Resource 1 The representative morphologic appearance of blasts in the APL group. The tumor cells are of medium to large size. The nuclei are usually round in the hypergranular type of APL (P1, P2, P4, P5), but pathological promyelocytes may have lobulated or cerebriform nuclei (P3, P6). The cytoplasm of hypergranular APL blasts can be characterized by intense azurophilic granulation (P1–P6). Pathological cells can contain Auer-roads (P4). The nuclei are distinctly lobulated and the cytoplasm contains sparsely azurophilic granulation in microgranular-type APL cases (P7, P8). (PDF 379 kb)
