# Supplementary material for: A novel flow cytometric method for enhancing acute promyelocytic leukemia screening by multidimensional dot-plots
Source: Ann Hematol. 2019 Mar 4;98(6):1413–20. doi: 10.1007/s00277-019-03642-w (PMC6511347; doi:10.1007/s00277-019-03642-w)

A novel flow cytometric method for enhancing acute promyelocytic leukemia screening by multidimensional dot plots

Annals of Hematology

Bettina Kárai, Mira Habók, Gyula Reményi, László Rejtő, Anikó Ujfalusi, János Kappelmayer, Zsuzsanna Hevessy

Corresponding author: Bettina Kárai M.D.

Department of Laboratory Medicine, Faculty of Medicine, University of Debrecen,

karai.bettina@med.unideb.hu

Online Resource 1

P-1

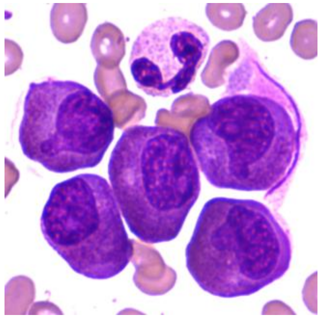

P-2

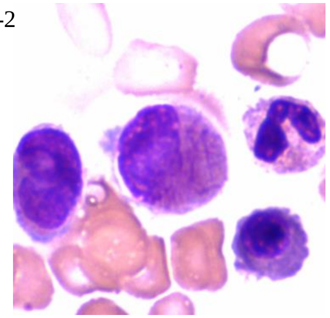

P-3

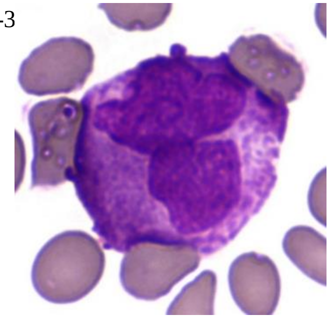

P-4

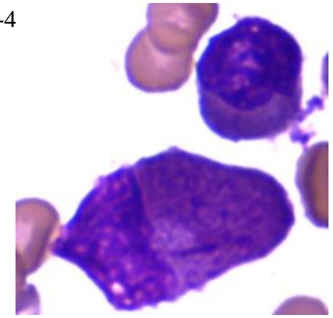

P-5

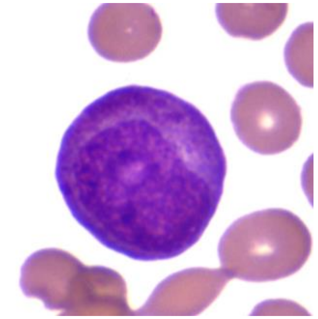

P-6

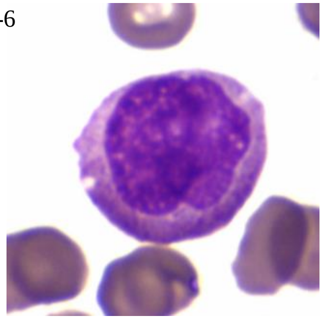

P-7

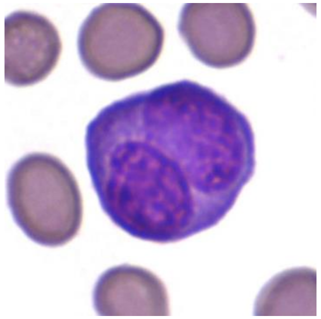

P-8

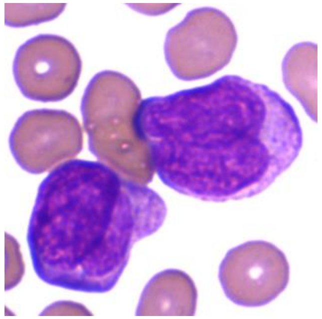

Supplement: Supplementary file 1 — The representative morphologic appearance of blasts in the APL group. The tumor cells are of medium to large size. The nuclei are usually round in the hypergranular type of APL (P1, P2, P4, P5), but pathological promyelocytes may have lobulated or cerebriform nuclei (P3, P6). The cytoplasm of hypergranular APL blasts can be characterized by intense azurophilic granulation (P1–P6). Pathological cells can contain Auer-roads (P4). The nuclei are distinctly lobulated and the cytoplasm contains sparsely azurophilic granulation in microgranular-type APL cases (P7, P8). (PDF 379 kb) [file 277_2019_3642_MOESM1_ESM.pdf]
